# Prevalence and factors associated with tobacco and cannabis co-use in France: Results from a national representative survey

**DOI:** 10.1016/j.dadr.2025.100381

**Published:** 2025-09-16

**Authors:** Tangui Barré, Vincent Di Beo, Camelia Protopopescu, Emmanuel Lahaie, Raphaël Andler, Viêt Nguyen-Thanh, Anne Pasquereau, Patrizia Carrieri, François Beck

**Affiliations:** a Aix-Marseille Univ, Inserm, IRD, SESSTIM, Sciences Economiques & Sociales de la Santé & Traitement de l’Information Médicale, ISSPAM, Marseille, France; b Santé Publique France, Saint-Maurice, France; c Centre de Recherche en Épidémiologie et Santé des Populations (CESP), Inserm U1018, Université Paris-Saclay, Université Paris-Sud, Université Versailles Saint-Quentin (UVSQ), Villejuif, France

**Keywords:** Tobacco, Cannabis, France, Co-use, Socioeconomic

## Abstract

**Background and aims:**

Tobacco use is a leading global risk factor for premature mortality. Cannabis-related harms are well documented, and its co-use with tobacco may hinder smoking cessation efforts. Moreover, tobacco use may amplify certain cannabis-related harms. To inform targeted interventions, we provided estimates of tobacco and cannabis co-use prevalence and correlates in France.

**Methods:**

Using data from a 2021 nationwide representative sample of French population aged 18–64 years, we estimated the prevalence of tobacco and cannabis co-use. Co-use was defined as reporting daily cigarette smoking and at least monthly cannabis use. We used multinomial logistic regression models to identify factors associated with co-use.

**Results:**

Among the 18,288 study participants, 71.8 % reported no use of cannabis or tobacco (‘no-use’ group), 22.3 % tobacco mono-use, 1.4 % cannabis mono-use, and 4.5 % co-use of both substances. Individuals who co-used reported a more frequent cannabis use than those who mono-used cannabis. Having financial difficulties was significantly associated with co-use, compared to tobacco and cannabis mono-use. After multivariable adjustment, the odds of co-use compared with ‘no-use’ was 2.3 times higher for participants with a poor health status, and 3.0 times higher for those with financial difficulties. Reporting a major depressive episode and unhealthy alcohol use were also significantly associated with co-use.

**Conclusions:**

The clinical management of individuals who co-use tobacco and cannabis should include comprehensive assessments of physical and mental health, as well as alcohol consumption. Interventions to reduce co-use-related harms should also address the adverse social conditions experienced by this population.

## Highlights

• Five percent of French adults co-use tobacco and cannabis.
• Most French adults who use cannabis use also tobacco.
• Tobacco and cannabis co-use is associated with a lower socioeconomic status.

## 1 Introduction

Tobacco smoking was the leading risk factor for premature mortality among males in 2019 globally (GBD, 2019 Risk Factors Collaborators, 2020). Despite a decrease in prevalence since 1990 (GBD, 2019 Tobacco Collaborators, 2021), most countries in Europe had between 10 % and 20 % of disability-adjusted life-years (DALYs) attributable to smoking in 2019 (GBD, 2019 Risk Factors Collaborators, 2020). The global DALYs of cannabis use disorder (CUD) in 2019 summed up 0.69 million (Shah et al., 2024, Shao et al., 2023). Based on the most recent surveys, last-year cannabis use among the EU population aged 15–34 is estimated at 15.0 % (European Union Drugs Agency, 2024). Unlike tobacco, increasing trends have been observed for cannabis use prevalence in Europe between 2010 and 2019 (Manthey et al., 2021), but the burden of cannabis use disorder (CUD) did not seem to increase in the last decades, with notably stable high age-standardized DALYs rate observed in Western Europe, especially among young adults (Shah et al., 2024, Shao et al., 2023). Cannabis use may cause adverse health effects even among those with no CUD. It has been associated with impairments in cognition and motivation, as well as with mood and psychotic disorders (Hoch et al., 2024). Efforts to reduce tobacco and cannabis smoking rates at global and European levels must therefore be pursued.

In France, the prevalence of daily tobacco smoking in adults has slightly dropped in the last decades from 30.0 % in 2000 to 25.3 % in 2021 (Pasquereau et al., 2022), while past-month cannabis use remained stable around 6 % between 2014 and 2021 (Le Nézet et al., 2022). In France, tetrahydrocannabinol-containing products are illegal and their possession is criminalized (Massin et al., 2013), except within the framework of an ongoing national medical cannabis experimental project initiated in 2021, which aims to assess the feasibility of providing medical cannabis to individuals with chronic severe conditions not adequately alleviated by other treatments.

People who co-use tobacco and cannabis (defined as those who use both substances either separately or simultaneously in a recent period) represent a particular target in this broad objective of reducing tobacco- and cannabis-related harms (Sumodhee et al., 2024), as both substances are strongly intertwined in several respects (Lemyre et al., 2019). The use of cannabis is associated with an increased risk of nicotine dependence and of tobacco smoking frequency (Agrawal et al., 2011, Agrawal et al., 2008a, Agrawal et al., 2008b, Rubinstein et al., 2014, Wang et al., 2016). In parallel, tobacco co-use may be associated with CUD (Hindocha et al., 2015). Cannabis use is also associated with poorer tobacco cessation outcomes (ANRS CO13 HEPAVIH Study Group, 2021, Voci et al., 2024, Voci et al., 2020, Weinberger et al., 2018), while the reciprocal is not consistently supported (McClure et al., 2020). Lastly, co-use may pose additive risk for toxicant exposure (Meier and Hatsukami, 2016, Smith et al., 2019bSmith et al., 2019b), and has been associated with higher levels of externalizing mental health problems than use of only one of the two substances (Do et al., 2024). While the causal directions are unclear, the associations between depression and tobacco use (Fluharty et al., 2017, Yuan et al., 2020) or cannabis use (Churchill et al., 2025) are both well documented. People who co-use are therefore at high risk of depression (Nguyen et al., 2023). The urgent need for treatment addressing co-use of tobacco and cannabis has consequently been highlighted (Nguyen et al., 2024).

To better understand this public health issue and guide targeted interventions, the first step is the estimation of the prevalence of co-use at the population level (Hindocha and McClure, 2021). In a 2017–2018 US nationally representative cohort of adults, Cohn and Chen found that 33 % of those who used tobacco in the past month also used cannabis in the past month, the co-use level approaching 50 % in 18–24 years adults (Cohn and Chen, 2022). However, while substance-related practices differ from the US (Hindocha et al., 2016), there are few data reporting such prevalence of co-use in Europe. In 2022–2023 in Germany, it has been estimated that 10 % of people currently using tobacco co-used cannabis in the past year (Kotz et al., 2024). Furthermore, identifying the profile of people who co-use may help targeting interventions to reduce harms related to such practices.

In this study, we aimed to estimate the prevalence of tobacco and cannabis co-use in France, identify associated factors, and compare levels of use between individuals who consume both substances and those who consume only one.

## 2 Material and methods

### 2.1 Design

From 11 February to 15 December (excluding a summer break from 19 July to 22 August) 2021, the French National Public Health Agency (*Santé Publique France*) implemented the 2021 French Health Barometer survey (Soullier et al., 2022). This national cross-sectional survey investigates health-seeking behaviours and perceptions, and has been regularly conducted by *Santé Publique France* since 1992.

Potential participants were contacted by telephone. To do this, landline and mobile telephone numbers were randomly generated to ensure the inclusion of individuals with unlisted numbers. For landlines, a two-stage random sampling process was used as follows: after random number generation, a single eligible individual within each household contacted was randomly selected. For mobile lines, the individual who answered the call was interviewed. The questionnaire was administered using the computer-assisted telephone interviewing (CATI) system, where the interviewer conducts the interview by phone while following a pre-established script displayed on a computer screen.

Participants had to provide consent to participate in the survey and to allow the processing of their personal data, including health-related information. As per French law, approval from a national ethics committee was not required, as the survey was not legally classified as research involving human subjects. Participants did not received compensation for their participation.

### 2.2 Participants

The survey included French-speaking individuals aged 18–75 years residing in mainland France. Residents of collective dwellings (e.g., nursing homes, homeless shelters) and institutions (e.g., hospitals) were excluded from the target population. For the present study, we excluded people aged ≥ 65 years, as cannabis-related questions were not asked in this age group.

### 2.3 Sample size

The French Health Barometer of *Santé Publique France* is a multi-thematic survey. A total of 24,514 participants were included in the study. This sample size had been calculated in advance to ensure that there would be at least 1000 participants in each the 12 regions of mainland France (the Provence-Alpes-Côte d’Azur and Corsica regions were combined into one region), allowing sufficient precision for regional estimates of the main variables of interest (Soullier et al., 2022).

### 2.4 Data collection

The average time required to complete the questionnaire was 36 min. Sociodemographic data collected included variables such as age, sex, employment status, highest educational qualification, self-perceived household economic status, and urban unit size (a French statistical measure of contiguously built-up areas) (Institut National de la Statistique et des Etudes Economiques, 2023).

Items related to the presence of a major depressive episode from the World Health Organization Composite International Diagnostic Interview Short-Form (CIDI-SF) were asked (Kessler et al., 1998, Léon et al., 2023). Overall health state was assessed by the question “How is your general state of health?” (five possible answers). Alcohol use was assessed with the AUDIT-C (Bush et al., 1998).

Participants were asked separately how many industrial and roll-your-own tobacco cigarettes they habitually smoke. They could report these numbers per day, week, month or year. Self-reported time to the first cigarette of the day was also collected to compute the Heavy Smoking Index (HSI) (Etter et al., 1999, Heatherton et al., 1989). The frequency of electronic cigarette use was collected, with three possible answers (daily, at least once a week, or less than once a week).

The cannabis section was introduced by the interviewer as follows: “We will now talk about cannabis, that is, hashish, marijuana, weed, joints, or shit. I would like to remind you that your answers will remain completely anonymous”. Cannabis use was assessed using the verb “to consume”, irrespective of the mode of administration. Past-month use of cannabis was assessed among those reporting past-year use, through the question “Did you do it [consuming cannabis] in the last 30 days? (yes, no)”. In accordance with the International Cannabis Toolkit (Lorenzetti et al., 2022), if the answer was positive, participants were asked “How many times in the last 30 days have you used cannabis?”, with the indication for the interviewer “Please note that we're talking about the number of days. For people who declare that they use cannabis several times a day, or every day, code 30.”

### 2.5 Data weighting

To enhance the representativeness of the estimates, data were weighted. The final weights were derived using a two-step process. First, the initial weighting accounted for the probability of inclusion, which depended on the number of eligible individuals and the number of telephone lines (landline or mobile) within each contacted household. Margin calibration was then applied, which adjusted for the structure of the general population in mainland France, based on the following variables: sex crossed with age in ten-year bands, household composition, highest educational qualification, region of residence, and size of the urban unit (using 2020 data (Institut National de la Statistique et des Etudes Economiques, 2021a)). The margin calibration process produced the final weights used in the analyses.

### 2.6 Outcomes

Daily tobacco use is a widely used marker for tobacco-related behaviors (Birge et al., 2018, GBD 2015 Tobacco Collaborators, 2017, Jackson et al., 2024). Moreover, people who smoke tobacco on a daily basis are likely to identify themselves as smokers (Leas et al., 2015). To ease future inter-country comparisons, we therefore considered daily tobacco use (smoking at least one tobacco cigarette of any type per day) as the outcome. Electronic cigarette use was not considered as “tobacco use”. For cannabis use, we considered past-month use, as commonly used in the literature (Cohn and Chen, 2022, Manthey et al., 2021). This choice is also in agreement with other recommended measures based on days of use per month (Lorenzetti et al., 2022). According to those two variables, we designed tobacco and cannabis use status as a four-category variable: tobacco mono-use (i.e. daily cigarette smoking and no past-month cannabis use), cannabis mono-use (i.e. past-month cannabis use and no daily cigarette smoking), co-use (i.e. daily cigarette smoking and past-month cannabis use), and no use (i.e. no daily cigarette smoking and no past-month cannabis use).

### 2.7 Explanatory and descriptive variables

Age was categorized in four categories (18–30, 31–44, 45–54, and 55–64 years). Employment status was dichotomized into two categories: having a job or not. Education level was classified into three categories: ‘<  upper secondary school certificate’, ‘upper secondary school certificate’, and ‘>  upper secondary school certificate’. Self-perceived household economic status was evaluated with the following question: “Presently, would you say that, in your household, financially speaking…” with response options including: ‘You are comfortable’, ‘You are ok’, ‘You just get by’, ‘It’s difficult to make ends meet’, ‘You can’t manage without going into debt (or using consumer credit)’ (Institut National de la Statistique et des Etudes Economiques, 2021b). The two last modalities were merged. Urban area size was categorized into small, medium, and large urban units, corresponding to three population ranges: < 20,000; 20,000–199,999; and ≥ 200,000 inhabitants, respectively.

For self-perceived general health status, ‘poor’ and ‘very poor’ answers were merged. Based on the CIDI-SF, the presence of a major depressive disorder was characterized by two weeks of dysphoric mood (lasting at least most of the day, at least almost every day) or two weeks of anhedonia (lasting at least most of the day, at least almost every day), plus, on the same period, by the presence of at least three secondary symptoms, and one impairment (Sapinho et al., 2008). Unhealthy alcohol use was characterized by an AUDIT-C score ≥ 3 for women, and ≥ 4 for men. Current electronic cigarette use was classified as either ‘daily’ or ‘not daily’. A moderate or high nicotine dependence was characterized by an HSI score ≥ 4 (Chabrol et al., 2005, de Leon et al., 2003, Diaz et al., 2005).

### 2.8 Statistical analyses

The weighted prevalence with 95 % confidence interval (CI) of each tobacco and cannabis use category — tobacco mono-use, cannabis mono-use, co-use, and no-use — was assessed in the whole study sample. The prevalence of tobacco and cannabis use categories was compared between the modalities of each descriptive variable (Rao-Scott Chi-square test accounting for the weighting design).

To identify factors associated with co-use as compared to no-use, we used multinomial logistic regression models. Associations were assessed using odds ratios (OR) in bivariable analyses, and adjusted odds ratios (aOR) in the multivariable analysis. Only explanatory variables with a liberal p-value < 0.20 in the bivariable analyses were considered eligible for the multivariable models (Hosmer and Lemeshow). The final multivariable model was built using a backward stepwise selection procedure. The likelihood ratio test (p < 0.05) was used to define which variables to keep in the final model. We then ran this final model while changing the reference group to identify factors associated with co-use as compared to tobacco mono-use and cannabis mono-use, respectively. All analyses used weighted data.

We conducted two sensitivity analyses. In a first one, we tested the robustness of our results when the definition of tobacco use was extended to past-month cigarette smoking (rather than daily cigarette smoking). In a second one, we verified that electronic cigarette use was not a confounding factor. To do so, we conducted the analyses without including electronic cigarette use as a potential explanatory variable.

Analyses were performed with Stata software version 17.0 (StataCorp LP, College Station, TX, USA).

## 3 Results

### 3.1 Prevalence of tobacco and cannabis co-use in French adults

Of the 18,288 participants, 71.8 % (95 %CI: [71.0–72.7]) reported no use of either substance (reference group), 22.3 % [21.5–23.1] tobacco mono-use, 1.4 % [1.2–1.6] cannabis mono-use, and 4.5 % [4.1–5.0] co-use. The prevalence of co-use was significantly higher in men, in people aged 18–30 years, in those with no job, with lower education, with financial difficulties, in people with a major depressive disorder, with a poorer self-perceived general health state, with an unhealthy alcohol use, and those using an electronic cigarette daily (Table 1).

**Table 1:** Study sample characteristics according to tobacco and cannabis use status (weighted values, n = 18,288).

	**All study sample**	**No use (71.8 %)**	**Tobacco mono-use**^a^**(22.3 %)**	**Cannabis mono-use**^a^**(1.4 %)**	**Tobacco and cannabis**^a^**co-use (4.5 %)**		**Prevalence of tobacco and cannabis co-use**
	**%**	**%**	**%**	**%**	**%**	**p-value**^b^	**% [95 % CI]**^c^
**Sex**						< 0.001	
Men	48.9	47.2	49.2	69.7	69.3		6.4 [5.7–7.1]
Women	51.1	52.8	50.8	30.3	30.7		2.7 [2.2–3.2]
**Age** (in years)						< 0.001	
18–30	25.2	24.2	22.1	61.8	46.3		8.3 [7.2–9.4]
31–44	31.9	31.0	34.4	26.6	35.4		5.0 [4.2–5.9]
45–54	23.0	23.0	25.7	7.9	14.1		2.8 [2.1–3.5]
55–64	19.9	21.8	17.9	3.7	4.2		0.9 [0.6–1.3]
**Having a job**						< 0.001	
No	33.0	31.3	35.1	42.3	45.6		6.3 [5.4–7.2]
Yes	67.0	68.7	64.9	57.7	54.4		3.7 [3.2–4.1]
**Educational level**						< 0.001	
<Upper secondary school certificate	40.0	34.8	56.1	15.6	51.1		5.8 [4.9–6.7]
Upper secondary school certificate	22.2	22.4	20.1	36.3	23.7		4.8 [4.0–5.6]
> Upper secondary school certificate	37.8	42.7	23.8	48.1	25.2		3.0 [2.6–3.5]
**“Presently, would you say that in your household, financially speaking…?”**						< 0.001	
You are comfortable	20.4	22.7	14.0	30.4	13.1		2.9 [2.2–3.6]*
You are ok	46.1	48.6	40.5	45.7	33.4		3.3 [2.8–3.8]*
You just get by	20.7	18.9	25.2	15.2	29.7		6.5 [5.3–7.7]
It’s difficult to make ends meet/You can’t manage without going into debt (or using consumer credit)	12.7	9.8	20.2	8.7	23.8		8.5 [6.8–10.2]
**Urban unit size** (number of inhabitants)						< 0.001	
< 20 000	38.5	38.3	41.3	24.7	32.6		3.8 [3.2–4.5]*
20 000–199 999	18.3	17.7	20.3	18.7	18.3		4.5 [3.5–5.5]*^#^
≥ 200 000	43.2	44.1	38.4	56.7	49.0		5.2 [4.4–5.9]^#^
**Major depressive episode**^d^						< 0.001	
No	89.3	91.0	85.5	83.6	82.6		4.2 [3.7–4.6]
Yes	10.7	9.0	14.5	16.4	17.4		7.4 [5.7–9.1]
**Self-reported health state**						< 0.001	
Very good	28.1	29.3	24.4	30.6	25.9		4.2 [3.4–5.0]*
Good	42.4	43.9	38.1	46.1	37.5		4.0 [3.4–4.6]*
Quite good	22.8	21.4	27.1	16.7	24.7		4.9 [4.0–5.8]*
Poor / Very poor	6.8	5.3	10.4	6.6	11.9		7.9 [5.6–10.3]
**Unhealthy alcohol use**^e^						< 0.001	
No	42.9	46.6	36.4	22.2	22.0		2.3 [1.8–2.8]
Yes	57.1	53.4	63.6	77.8	78.0		6.1 [5.5–6.8]
**Daily electronic cigarette use**						< 0.001	
No	94.2	95.4	91.6	84.2	91.6		4.4 [4.0–4.8]
Yes	5.8	4.6	8.4	15.8	8.4		6.6 [4.5–8.7]
**Nicotine dependence**^f^, ^g^							
Low	95.9	-	84.9	-	83.7		
Moderate or high	4.1		15.1		16.3		
**Cigarettes smoked per day**^g^						-	
< 10	88.2	-	55.1	-	61.8		
11–20	9.3		35.5		28.9		
21–30	1.8		6.5		6.4		
> 30	0.8		2.8		2.9		
**Days of cannabis use per month**^g^						-	
< 20	97.8	-	-	84.0	56.2		
≥ 20	2.2			16.0	43.8		

CI, confidence interval.

Data were weighted according to the probability of inclusion, followed by a margin calibration using the sex variable crossed with age in decennial bands, household size, educational qualification, region of residence and size of urban unit (based on 2020 data (Institut National de la Statistique et des Etudes Economiques, 2021a)).

a Tobacco use is defined as daily cigarette smoking. Cannabis use is defined as past-month use.

b Rao-Scott Chi-square test (null hypothesis of no difference between the four groups according to tobacco and cannabis use status).

c Within each variable, prevalence categories sharing the same symbol are not significantly different from each other, whereas categories without common symbols are significantly different from each other. Within each variable, categories without any symbol are significantly different from all other categories (Rao-Scott Chi-square test).

d According to the World Health Organization Composite International Diagnostic Interview Short-Form (CIDI-SF) (Kessler et al., 1998, Léon et al., 2023).

e Unhealthy alcohol use was defined as an AUDIT-C score ≥ 3 for women, and ≥ 4 for men (Bush et al., 1998).

f Nicotine dependence was assessed with the Heavy Smoking Index (HSI), using a cut-off of ≥ 4 for moderate or high dependence (Etter et al., 1999, Heatherton et al., 1989).

g Used only for descriptive analyses.

Among all people using tobacco, 16.9 % co-used cannabis. Among all people using cannabis, 80.0 % co-used tobacco. People co-using tobacco and cannabis were more likely to report daily or near-daily (≥20 days per month) cannabis use than those who mono-use cannabis (p < 0.001), and reported a greater number of cannabis consumption days per month (median [interquartile range] of 10 [3−30] vs. 3 [2−10], (p < 0.001)). However, people who co-use were no more likely than those who mono-use tobacco to have a moderate or high nicotine dependence (p = 0.628), nor to smoke a greater number of cigarettes per day (p = 0.168).

### 3.2 Factors associated with tobacco and cannabis co-use

After multivariable adjustment, and as compared with no use, being a man was associated with both tobacco and cannabis co-use and cannabis mono-use. Younger age, having a major depressive episode, unhealthy alcohol use, and daily electronic cigarette use were associated with the three tobacco and cannabis use categories, as compared with no use. A lower educational level, financial difficulties, and a poor health status were associated with both tobacco mono-use and tobacco and cannabis co-use, whereas having an educational level higher than upper secondary school certificate (vs. lower than upper secondary school certificate) was associated with cannabis mono-use. Having no job was associated with co-use (Table 2).

**Table 2:** Factors associated with tobacco and cannabis use status (multivariable multinomial regression, with no-use as the reference group, weighted analyses, n = 18,036).

	**Tobacco mono-use**^a^		**Cannabis mono-use**^a^		**Tobacco and cannabis co-use**^a^	
	**aOR [95 % CI]**	**p-value**	**aOR [95 % CI]**	**p-value**	**aOR [95 % CI]**	**p-value**
**Sex**						
Men	1.1 [1.0–1.2]	0.062	2.7 [1.9–3.7]	< 0.001	3.0 [2.4–3.7]	< 0.001
Women (ref.)	1		1		1	
**Age** (in years)						
18–30	1.5 [1.3–1.8]	< 0.001	10.6 [5.2–21.7]	< 0.001	14.9 [9.3–24]	< 0.001
31–44	1.8 [1.5–2.0]	< 0.001	4.0 [1.9–8.6]	< 0.001	8.6 [5.3–14.1]	< 0.001
45–54	1.5 [1.3–1.8]	< 0.001	1.9 [0.8–4.7]	0.146	3.7 [2.2–6.3]	< 0.001
55–64 (ref.)	1		1		1	
**Having a job**						
No	1.0 [0.9–1.1]	0.873	1.4 [1.0–2.0]	0.062	1.5 [1.2–1.9]	0.001
Yes (ref.)	1		1		1	
**Educational level**						
<Upper secondary school certificate	2.6 [2.3–3.0]	< 0.001	0.5 [0.3–0.8]	0.004	2.4 [1.9–3.0]	< 0.001
Upper secondary school certificate	1.5 [1.3–1.7]	< 0.001	0.9 [0.7–1.3]	0.697	1.2 [0.9–1.5]	0.213
> Upper secondary school certificate (ref.)	1		1		1	
**“Presently, would you say that in your household, financially speaking…?”**						
You are comfortable (ref.)	1		1		1	
You are ok	1.2 [1.1–1.4]	0.005	0.9 [0.6–1.3]	0.487	1.3 [1.0–1.8]	0.084
You just get by	1.7 [1.5–2.0]	< 0.001	0.7 [0.5–1.2]	0.241	2.6 [1.9–3.5]	< 0.001
It’s difficult to make ends meet/You can’t manage without going into debt (or using consumer credit)	2.2 [1.8–2.6]	< 0.001	0.8 [0.4–1.7]	0.618	3.0 [2.1–4.3]	< 0.001
**Major depressive episode**^b^						
No (ref.)	1		1		1	
Yes	1.5 [1.3–1.7]	< 0.001	1.9 [1.2–3.0]	0.004	1.6 [1.2–2.2]	0.001
**Self-reported health status**						
Very good (ref.)	1		1		1	
Good	1.0 [0.9–1.2]	0.694	1.2 [0.8–1.6]	0.398	1.1 [0.8–1.4]	0.613
Quite good	1.3 [1.1–1.4]	0.002	0.9 [0.5–1.4]	0.623	1.4 [1.0–1.9]	0.040
Poor / Very poor	1.6 [1.3–2.0]	< 0.001	1.9 [0.9–4.1]	0.085	2.3 [1.5–3.6]	< 0.001
**Unhealthy alcohol use**^c^						
No (ref.)	1		1		1	
Yes	1.5 [1.3–1.6]	< 0.001	2.4 [1.6–3.6]	< 0.001	2.6 [2.0–3.3]	< 0.001
**Daily electronic cigarette use**						
No (ref.)	1		1		1	
Yes	1.7 [1.4–2.1]	< 0.001	3.6 [2.4–5.5]	< 0.001	1.6 [1.1–2.3]	0.023

aOR, adjusted odds ratio; CI, confidence interval.

Data were weighted according to the probability of inclusion, followed by a margin calibration using the sex variable crossed with age in decennial bands, household size, educational qualification, region of residence and size of urban unit (based on 2020 data (Institut National de la Statistique et des Etudes Economiques, 2021a)).

a Tobacco use is defined as daily cigarette smoking. Cannabis use is defined as past-month use.

b According to the World Health Organization Composite International Diagnostic Interview Short-Form (CIDI-SF) (Kessler et al., 1998, Léon et al., 2023).

c Unhealthy alcohol use was defined as an AUDIT-C score ≥ 3 for women, and ≥ 4 for men (Bush et al., 1998).

For the same model, we also examined associations when comparing tobacco and cannabis co-use with mono-uses. As compared to tobacco mono-use, being a man, being between 18 and 30 years, having no job, reporting financial difficulties (‘just get by’) and unhealthy alcohol use were associated with co-use. As compared to cannabis mono-use, lower educational level, reporting financial difficulties, and no daily use of electronic cigarette were associated with co-use (Supplementary Table 1).

In the sensitivity analysis, when considering past-month (instead of daily) cigarette smoking, prevalence of tobacco and cannabis co-use increased to 5.3 % [4.8–5.7], and similar patterns of prevalence levels within variable categories were observed (Supplementary Table 2). Regarding factors associated with tobacco and cannabis use status, only minor changes were observed as compared to the main analysis (the highest educational level was no longer associated with cannabis mono-use vs. no use; daily electronic cigarette use was no longer inversely associated with co-use vs. cannabis mono-use, p = 0.077 (Supplementary Table 3 and Supplementary Table 4**).** When removing electronic cigarette use from the analyses, the results remained unchanged (data not shown).

## 4 Discussion

This study put into evidence three main results, which may have major repercussions on care for people co-using cannabis and tobacco. First, those who co-used tobacco and cannabis reported a more frequent cannabis use than those who mono-used cannabis. Second, a lower socioeconomic status was associated with co-use as compared to all other groups (no-use, tobacco mono-use, and cannabis mono-use). Third, reporting a major depressive episode and unhealthy alcohol use were both associated with all tobacco and cannabis use categories, as compared with no-use. Unhealthy alcohol use was also associated with co-use as compared to tobacco mono-use.

The prevalence of co-use we found within French adults who smoke tobacco cigarettes daily (17 %) was higher than the one reported in Germany (10 %) (Kotz et al., 2024), reflecting the slightly higher prevalence of cannabis use in France than in Germany (European Union Drugs Agency, 2024). We previously showed that while tobacco screening is often implemented by French general practitioners, cannabis screening is far less common (Barré et al., 2024). The fact that more than one in seven individuals who daily used tobacco also used cannabis indicates that screening for cannabis use should be encouraged in people who daily use tobacco to improve the clinical management of co-use, in particular for people aged 18–44 years old.

The relationships between tobacco use and low socioeconomic status have been widely documented (Casetta et al., 2017, Hitchman et al., 2014), including in France (Pasquereau et al., 2023, Peretti-Watel et al., 2009b). The same is true for cannabis use (Jeffers et al., 2021, Legleye et al., 2016, Mattingly et al., 2024). Here, we showed that people who co-used were underprivileged as compared to people with no use and those with mono-use. This finding is particularly relevant, as individuals with low socioeconomic status often encounter multiple challenges that can hinder their efforts to quit smoking (van Wijk et al., 2019). Cannabis co-use in people who use tobacco has been associated with lower motivation to quit tobacco (Twyman et al., 2016). People who co-use are therefore likely to face cannabis-related barriers, such as persistent exposure to smoking cues or enhanced sensitivity to nicotine (Lemyre et al., 2019), in addition to socioeconomically related barriers. When considering tobacco cessation interventions in this population, both types of barriers should therefore be addressed by specific tailoring (Kock et al., 2019, Sumodhee et al., 2024).

Tobacco price increases are expected to reduce socioeconomic health inequalities (Brown et al., 2014, Hill et al., 2014, Smith et al., 2020). In France, while some disadvantaged smokers may be less sensitive to such increases (Peretti-Watel et al., 2009a, Peretti-Watel and Constance, 2009), recent data indicate that the desire to quit and the intention to quit within the next six months were associated with reporting a less comfortable financial situation (Guignard et al., 2023). However, despite existing data from the U.S. suggesting independence of the relationship between price elasticity for the two substances (Cooper et al., 2023), we do not know specifically how people who co-use tobacco and cannabis in France respond to tobacco price increases.

We found that people who co-used tobacco and cannabis reported more frequent cannabis use, suggesting higher risks of problematic use, including CUD (Callaghan et al., 2020, Fischer et al., 2017, Hindocha et al., 2015). These findings support the importance of systematically addressing tobacco smoking in the treatment of CUD. Moreover, as people who co-use generally consume equal or greater amounts of each substance than those who mono-use, the former are also exposed to greater amounts of carcinogens and respiratory toxins (Graves et al., 2020, Janssen et al., 2024, Tashkin and Roth, 2019).

Thus, in case of co-use, promoting alternative nicotine and cannabis delivery systems may therefore support cessation and serve as effective combustion-related harm-reduction strategies. Electronic cigarette use may be seen as a harm reduction strategy in some circumstances (Feeney et al., 2022), such as among hard-to-treat people who smoke tobacco (a designation that may include people who co-use tobacco and cannabis) (Stevens and Sherman, 2022). Regarding smokeless delivery of cannabis, the use of vaporizer can reduce the emission of carbon monoxide, chronic respiratory symptoms, and exposure to several toxins while producing similar subjective effects (Chaiton et al., 2021, MacCallum et al., 2024). Acceptability and safety of such devices should be investigated in this population (MacCallum et al., 2025).

Using alternative delivery systems may help break the habit - common in Europe (Hindocha et al., 2016) - of mixing tobacco and cannabis. In France in 2020, 94 % of past-year cannabis users smoked it in the form of a joint at their last use, and 95 % of them mixed it with tobacco (Santé Publique France, 2021). This practice is linked to moderate or high nicotine dependence, and elevated risks for cannabis problems (Jayakumar et al., 2019). Separating both substances could weaken overlapping cues and allow people to quit one substance without needing to quit both.

People who experienced a major depressive episode had an increased odds of tobacco or cannabis mono-use, and of tobacco and cannabis co-use, as compared with no use of either substance. This is in line with findings from a study in US adults (Nguyen et al., 2023). However, we found no significant association with major depressive disorder between tobacco and cannabis mono-use and co-use, possibly because of limited number of people who use and co-use cannabis in our sample. Further longitudinal studies focusing on this topic are needed to explore the directionality of such associations, and to determine the extent to which tobacco and cannabis use may act as additive risk factors for depression.

Lastly, we found that people who reported unhealthy alcohol use had an increased odds of tobacco and cannabis co-use, over no use of these substances. Besides, they had also an increased odds of co-use over tobacco mono-use. Co-use of alcohol and cannabis may be more harmful than the use of either substance alone (Yurasek et al., 2017). Given that alcohol and cannabis can function as both substitutes and complements (Coelho et al., 2023, Risso et al., 2020), changes in the use of one substance may unintentionally impact the other one.

These findings underscore the importance of substance use and psychiatric disorder screening when addressing tobacco and/or cannabis use cessation/reduction, particularly in the light of evidence relating cannabis use, depression and alcohol use to poorer tobacco cessation outcomes (van Amsterdam and van den Brink, 2023, Weinberger et al., 2017).

The main strength of our study lies in its design, which allowed for national estimates of the prevalence for tobacco and cannabis co-use in France. By examining both mono- and co-use, the study offers a comprehensive view of common consumption patterns. Limitations include the definition of tobacco use, which was restricted to cigarette smoking; individuals who mixed cannabis with tobacco but do not smoke tobacco cigarettes may have been misclassified. As we did not assess such a practice, it may have led to an underestimation of tobacco use among people using cannabis. Cannabis use, which is illegal in France, may also have been underreported by individuals fearing legal action against them. Moreover, we based our analysis on a self-reported number of days of cannabis consumption, which did not take into account the quantity used by day or the potency of cannabis products used, which are known crucial factors to assess cannabis-related risks. We also supposed that cannabis was smoked, according to previous data from France (Santé Publique France, 2021), but it is likely that a minority of people who used cannabis used other routes of administration such as vaporizing or edibles. Lastly, the small number of people who mono-used cannabis may have limited the power to detect further associations.

To conclude, in French adults, we showed that tobacco use is very common within people who use cannabis, and that people who co-use both use cannabis more frequently than those who mono-use cannabis. As compared to those who mono-use either substance, people who co-use have a lower socioeconomic status, and may be more prone to unhealthy alcohol use. This is why the clinical management of individuals who co-use tobacco and cannabis requires thorough assessment of their physical and mental health, as well as their alcohol use. Interventions to reduce co-use-related harms should also address the challenging social environments faced by this population.

## Funding sources

Data collection was funded and supervised by Santé Publique France.

## Ethical statement

All procedures were performed in compliance with the World Medical Association Declaration of Helsinki.

Participants provided consent to participate in the survey and to allow the processing of their personal data, including health-related information. As per French law, approval from a national ethics committee was not required, as the survey was not legally classified as research involving human subjects.

## CRediT authorship contribution statement

**Emmanuel Lahaie:** Writing – review & editing, Investigation. **Camelia Protopopescu:** Writing – review & editing, Methodology. **Viêt Nguyen-Thanh:** Writing – review & editing, Investigation. **Raphaël Andler:** Writing – review & editing, Investigation. **Patrizia Carrieri:** Writing – review & editing, Validation, Methodology, Conceptualization. **Anne Pasquereau:** Writing – review & editing, Investigation. **François Beck:** Writing – review & editing, Investigation, Conceptualization. **Vincent Di Beo:** Writing – review & editing, Formal analysis. **Tangui Barré:** Writing – review & editing, Writing – original draft, Methodology, Conceptualization.

## Declaration of Competing Interest

The authors declare that they have no known competing financial interests or personal relationships that could have appeared to influence the work reported in this paper.

## Data Availability

The data that support the findings of this study are available on request from the corresponding author.

## Footnotes

Appendix A Supplementary data associated with this article can be found in the online version at doi:10.1016/j.dadr.2025.100381.
