# Supplementary material for: Prevalence and factors associated with tobacco and cannabis co-use in France: Results from a national representative survey
Source: Drug Alcohol Depend Rep. 2025 Sep 16;17:100381. doi: 10.1016/j.dadr.2025.100381 (PMC12494852; doi:10.1016/j.dadr.2025.100381)
Supplement: Supplementary file 1 — Supplementary material [file mmc1.docx]

**Supplementary Table 1. Factors associated with tobacco and cannabis co-use (multivariable multinomial regression, with tobacco mono-use and cannabis mono-use as the reference groups, respectively, weighted analyses, n=18,036)**

|  | **Tobacco and cannabis co-use vs. tobacco mono-use**^1^ | | **Tobacco and cannabis co-use vs. cannabis mono-use**^1^ | |
| --- | --- | --- | --- | --- |
| **Sex** | **aOR [95% CI]** | **p-value** | **aOR [95% CI]** | **p-value** |
| Men | 2.7 [2.1-3.4] | <0.001 | 1.1 [0.8-1.7] | 0.588 |
| Women (ref.) | 1 |  | 1 |  |
| **Age** (in years) |  |  |  |  |
| 18-30 | 9.9 [6.1-16.0] | <0.001 | 1.4 [0.6-3.3] | 0.430 |
| 31-44 | 4.9 [3.0-8.1] | <0.001 | 2.1 [0.9-5.2] | 0.097 |
| 45-54 | 2.4 [1.4-4.1] | 0.001 | 1.9 [0.7-5.4] | 0.204 |
| 55-64 (ref.) | 1 |  | 1 |  |
| **Having a job** |  |  |  |  |
| No | 1.5 [1.2-1.9] | 0.002 | 1.1 [0.7-1.6] | 0.785 |
| Yes (ref.) | 1 |  | 1 |  |
| **Educational level** |  |  |  |  |
| <Upper secondary school certificate | 0.9 [0.7-1.2] | 0.453 | 5.1 [2.9-8.9] | <0.001 |
| Upper secondary school certificate | 0.8 [0.6-1.0] | 0.085 | 1.3 [0.8-1.9] | 0.287 |
| > Upper secondary school certificate (ref.) | 1 |  | 1 |  |
| **“Presently, would you say that in your household, financially speaking…?”** |  |  |  |  |
| You are comfortable (ref.) | 1 |  | 1 |  |
| You are ok | 1.1 [0.8-1.5] | 0.676 | 1.5 [0.9-2.4] | 0.092 |
| You just get by | 1.5 [1.1-2.1] | 0.024 | 3.5 [1.9-6.1] | <0.001 |
| It’s difficult to make ends meet/You can’t manage without going into debt (or using consumer credit) | 1.4 [0.9-2.0] | 0.113 | 3.5 [1.7-7.4] | 0.001 |
| **Major depressive episode^2^** |  |  |  |  |
| No (ref.) | 1 |  | 1 |  |
| Yes | 1.1 [0.8-1.5] | 0.479 | 0.9 [0.5-1.4] | 0.573 |
| **Self-reported health state** |  |  |  |  |
| Very good (ref.) | 1 |  | 1 |  |
| Good | 1.0 [0.8-1.4] | 0.763 | 0.9 [0.6-1.4] | 0.710 |
| Quite good | 1.1 [0.8-1.5] | 0.558 | 1.6 [0.9-2.8] | 0.121 |
| Poor / Very poor | 1.4 [0.9-2.2] | 0.150 | 1.2 [0.5-2.8] | 0.691 |
| **Unhealthy alcohol use^3^** |  |  |  |  |
| No (ref.) | 1 |  | 1 |  |
| Yes | 1.8 [1.4-2.3] | <0.001 | 1.1 [0.7-1.7] | 0.786 |
| **Daily electronic cigarette use** |  |  |  |  |
| No (ref.) | 1 |  | 1 |  |
| Yes | 0.9 [0.6-1.3] | 0.632 | 0.4 [0.3-0.7] | 0.002 |

aOR, adjusted odds ratio; CI, confidence interval.

Data were weighted according to the probability of inclusion, followed by a margin calibration using the sex variable crossed with age in decennial bands, household size, educational qualification, region of residence and size of urban unit (based on 2020 data (Institut National de la Statistique et des Etudes Economiques, 2021a)).

^1^ Tobacco use is defined as daily cigarette smoking. Cannabis use is defined as past-month use.

^2^ According to the World Health Organization Composite International Diagnostic Interview Short-Form (CIDI-SF) (Kessler et al., 1998; Léon et al., 2023).

^3^ Unhealthy alcohol use was defined as an AUDIT-C score ≥3 for women, and ≥4 for men (Bush et al., 1998).

**Supplementary Table 2. Study sample characteristics according to tobacco and cannabis use status (sensitivity analysis, weighted values, n=18,288)**

|  | **All study sample** | **No use**  **(66.6%)** | **Tobacco  mono-use^1^**  **(27.6%)** | **Cannabis  mono-use^1^**  **(0.6%)** | **Tobacco  and cannabis^1^ co-use**  **(5.3%)** |  | **Prevalence of tobacco and cannabis co-use** |
| --- | --- | --- | --- | --- | --- | --- | --- |
|  | **%** | **%** | **%** | **%** | **%** | **p-value^2^** | **% [95% CI]**^3^ |
| **Sex** |  |  |  |  |  | <0.001 |  |
| Men | 48.9 | 47.2 | 48.7 | 74.6 | 68.8 |  | 7.4 [6.7-8.2] |
| Women | 51.1 | 52.8 | 51.3 | 25.4 | 31.2 |  | 3.2 [2.7-3.7] |
| **Age** (in years) |  |  |  |  |  | <0.001 |  |
| 18-30 | 25.2 | 23.3 | 24.6 | 57.0 | 49.1 |  | 10.3 [9.1-11.5] |
| 31-44 | 31.9 | 30.4 | 35.2 | 28.2 | 33.9 |  | 5.6 [4.8-6.5] |
| 45-54 | 23.0 | 23.5 | 23.9 | 8.7 | 13.1 |  | 3.0 [2.3-3.7] |
| 55-64 | 19.9 | 22.7 | 16.3 | 6.1 | 3.8 |  | 1.0 [0.6-1.4] |
| **Having a job** |  |  |  |  |  | <0.001 |  |
| No | 33.0 | 31.4 | 34.3 | 34.7 | 46.0 |  | 7.4 [6.4-8.3] |
| Yes | 67.0 | 68.6 | 65.7 | 65.3 | 54.0 |  | 4.3 [3.8-4.7] |
| **Educational level** |  |  |  |  |  | <0.001 |  |
| <Upper secondary school certificate | 40.0 | 35.4 | 50.8 | 18.9 | 45.6 |  | 6.0 [5.1-6.9]^a^ |
| Upper secondary school certificate | 22.2 | 22.2 | 21.1 | 39.8 | 25.1 |  | 6.0 [5.1-6.9]^a^ |
| > Upper secondary school certificate | 37.8 | 42.4 | 28.2 | 41.3 | 29.3 |  | 4.1 [3.6-4.6] |
| **“Presently, would you say that in your household, financially speaking…?”** |  |  |  |  |  | <0.001 |  |
| You are comfortable | 20.4 | 22.5 | 16.2 | 34.0 | 15.2 |  | 3.9 [3.2-4.7]^a^ |
| You are ok | 46.1 | 48.7 | 41.8 | 48.9 | 34.8 |  | 4.0 [3.4-4.6]^a^ |
| You just get by | 20.7 | 18.9 | 24.0 | 7.8 | 28.4 |  | 7.3 [6.1-8.5] |
| It’s difficult to make ends meet/You can’t manage without going into debt (or using consumer credit) | 12.7 | 9.9 | 18.1 | 9.2 | 21.6 |  | 9.0 [7.2-10.7] |
| **Urban unit size** (number of inhabitants) |  |  |  |  |  | <0.001 |  |
| <20 000 | 38.5 | 38.6 | 39.8 | 26.3 | 31.3 |  | 4.3 [3.6-5.0] |
| 20 000 - 199 999 | 18.3 | 17.6 | 20.1 | 18.4 | 18.4 |  | 5.3 [4.3-6.4]^a^ |
| ≥200 000 | 43.2 | 43.8 | 40.1 | 55.4 | 50.3 |  | 6.2 [5.4-6.9]^a^ |
| **Major depressive episode^4^** |  |  |  |  |  | <0.001 |  |
| No | 89.3 | 91.2 | 86.3 | 83.5 | 82.7 |  | 4.9 [4.4-5.4] |
| Yes | 10.7 | 8.8 | 13.7 | 16.5 | 17.3 |  | 8.6 [6.8-10.3] |
| **Self-reported health state** |  |  |  |  |  | <0.001 |  |
| Very good | 28.1 | 29.3 | 25.5 | 32.2 | 26.4 |  | 5.0 [4.1-5.8]^a^ |
| Good | 42.4 | 43.6 | 40.1 | 45.4 | 38.8 |  | 4.8 [4.2-5.5]^a^ |
| Quite good | 22.8 | 21.8 | 25.2 | 14.6 | 23.8 |  | 5.5 [4.6-6.5]^a^ |
| Poor / Very poor | 6.8 | 5.4 | 9.2 | 7.9 | 11.0 |  | 8.6 [6.2-11.0] |
| **Unhealthy alcohol use^5^** |  |  |  |  |  | <0.001 |  |
| No | 42.9 | 48.0 | 35.2 | 27.7 | 21.4 |  | 2.6 [2.1-3.1] |
| Yes | 57.1 | 52.0 | 64.8 | 72.3 | 78.6 |  | 7.2 [6.5-7.9] |
| **Daily electronic cigarette use** |  |  |  |  |  | <0.001 |  |
| No | 94.2 | 96.2 | 90.5 | 84.1 | 90.6 |  | 5.1 [4.6-5.5] |
| Yes | 5.8 | 3.8 | 9.5 | 15.9 | 9.4 |  | 8.7 [6.4-10.9] |
| **Nicotine dependence^6,7^** |  |  |  |  |  |  |  |
| Low | 95.9 | - | 87.7 | - | 85.8 |  |  |
| Moderate or high | 4.1 |  | 12.3 |  | 14.2 |  |  |
| **Cigarettes smoked per day^7^** |  |  |  |  |  | - |  |
| <10 | 88.2 | - | 63.5 | - | 66.7 |  |  |
| 11-20 | 9.3 |  | 28.9 |  | 25.2 |  |  |
| 21-30 | 1.8 |  | 5.3 |  | 5.5 |  |  |
| >30 | 0.8 |  | 2.3 |  | 2.5 |  |  |
| **Days of cannabis use per month^7^** |  |  |  |  |  | - |  |
| <20 | 97.8 | - | - | 82.8 | 60.3 |  |  |
| ≥20 | 2.2 |  |  | 17.2 | 39.7 |  |  |

CI, confidence interval.

Data were weighted according to the probability of inclusion, followed by a margin calibration using the sex variable crossed with age in decennial bands, household size, educational qualification, region of residence and size of urban unit (based on 2020 data (Institut National de la Statistique et des Etudes Economiques, 2021a)).

^1^ Tobacco use is defined in this sensitivity analysis as past-month cigarette smoking. Cannabis use is defined as past-month use.

^2^ Rao-Scott Chi-square test (null hypothesis of no difference between the four groups according to tobacco and cannabis use status).

^3^ Within each variable, prevalence categories sharing the same superscript letter are not significantly different from each other, whereas categories with different superscript letters are significantly different. Within each variable, categories without any superscript letter are significantly different from all other categories (Rao-Scott Chi-square test).

^4^ According to the World Health Organization Composite International Diagnostic Interview Short-Form (CIDI-SF) (Kessler et al., 1998; Léon et al., 2023).

^5^ Unhealthy alcohol use was defined as an AUDIT-C score ≥3 for women, and ≥4 for men (Bush et al., 1998).

^6^ Nicotine dependence was assessed with the Heavy Smoking Index (HSI), using a cut-off of ≥4 for moderate or high dependence (Etter et al., 1999; Heatherton et al., 1989).

^7^ Used only for descriptive analyses.

**Supplementary Table 3. Factors associated with tobacco and cannabis use status (sensitivity analysis, multivariable multinomial regression, with no-use as the reference group, weighted analyses, n=18,036)**

|  | **Tobacco mono-use^1^** | | **Cannabis mono-use^1^** | | **Tobacco and cannabis co-use^1^** | |
| --- | --- | --- | --- | --- | --- | --- |
|  | **aOR [95% CI]** | **p-value** | **aOR [95% CI]** | **p-value** | **aOR [95% CI]** | **p-value** |
| **Sex** |  |  |  |  |  |  |
| Men | 1.1 [1.0-1.2] | 0.236 | 3.1 [1.8-5.1] | <0.001 | 2.8 [2.3-3.5] | <0.001 |
| Women (ref.) | 1 |  | 1 |  | 1 |  |
| **Age** (in years) |  |  |  |  |  |  |
| 18-30 | 1.9 [1.6-2.2] | <0.001 | 6.5 [2.7-15.9] | <0.001 | 17.3 [11.1-27] | <0.001 |
| 31-44 | 2.0 [1.7-2.3] | <0.001 | 2.8 [1.1-7.2] | 0.039 | 9.1 [5.7-14.5] | <0.001 |
| 45-54 | 1.6 [1.4-1.8] | <0.001 | 1.2 [0.4-3.9] | 0.742 | 3.8 [2.3-6.3] | <0.001 |
| 55-64 (ref.) | 1 |  | 1 |  | 1 |  |
| **Having a job** |  |  |  |  |  |  |
| No | 1.0 [0.9-1.1] | 0.965 | 1.0 [0.6-1.7] | 0.934 | 1.5 [1.2-1.9] | <0.001 |
| Yes (ref.) | 1 |  | 1 |  | 1 |  |
| **Educational level** |  |  |  |  |  |  |
| <Upper secondary school certificate | 2.1 [1.9-2.3] | <0.001 | 0.7 [0.3-1.6] | 0.450 | 1.9 [1.5-2.4] | <0.001 |
| Upper secondary school certificate | 1.3 [1.2-1.4] | <0.001 | 1.3 [0.8-2.2] | 0.334 | 1.1 [0.8-1.3] | 0.640 |
| > Upper secondary school certificate (ref.) | 1 |  | 1 |  | 1 |  |
| **“Presently, would you say that in your household, financially speaking…?”** |  |  |  |  |  |  |
| You are comfortable (ref.) | 1 |  | 1 |  | 1 |  |
| You are ok | 1.1 [1.0-1.3] | 0.042 | 0.8 [0.4-1.3] | 0.341 | 1.2 [0.9-1.6] | 0.139 |
| You just get by | 1.5 [1.3-1.7] | <0.001 | 0.3 [0.1-0.8] | 0.017 | 2.2 [1.7-3.0] | <0.001 |
| It’s difficult to make ends meet/You can’t manage without going into debt (or using consumer credit) | 1.8 [1.5-2.2] | <0.001 | 0.8 [0.3-2.1] | 0.603 | 2.5 [1.8-3.5] | <0.001 |
| **Major depressive episode^2^** |  |  |  |  |  |  |
| No (ref.) | 1 |  | 1 |  | 1 |  |
| Yes | 1.4 [1.2-1.6] | <0.001 | 2.1 [1.0-4.3] | 0.038 | 1.7 [1.3-2.2] | <0.001 |
| **Self-reported health status** |  |  |  |  |  |  |
| Very good (ref.) | 1 |  | 1 |  | 1 |  |
| Good | 1.1 [1.0-1.2] | 0.262 | 1.1 [0.7-1.9] | 0.700 | 1.1 [0.9-1.4] | 0.383 |
| Quite good | 1.2 [1.0-1.3] | 0.013 | 0.7 [0.3-1.4] | 0.281 | 1.3 [1.0-1.8] | 0.046 |
| Poor / Very poor | 1.5 [1.2-1.9] | <0.001 | 2.2 [0.6-7.5] | 0.205 | 2.2 [1.5-3.4] | <0.001 |
| **Unhealthy alcohol use^3^** |  |  |  |  |  |  |
| No (ref.) | 1 |  | 1 |  | 1 |  |
| Yes | 1.6 [1.5-1.8] | <0.001 | 1.9 [1.1-3.3] | 0.023 | 2.8 [2.2-3.5] | <0.001 |
| **Daily electronic cigarette use** |  |  |  |  |  |  |
| No (ref.) | 1 |  | 1 |  | 1 |  |
| Yes | 2.4 [2.0-2.9] | <0.001 | 4.2 [2.2-7.8] | <0.001 | 2.3 [1.6-3.2] | <0.001 |

aOR, adjusted odds ratio; CI, confidence interval.

Data were weighted according to the probability of inclusion, followed by a margin calibration using the sex variable crossed with age in decennial bands, household size, educational qualification, region of residence and size of urban unit (based on 2020 data (Institut National de la Statistique et des Etudes Economiques, 2021a)).

**^1^** Tobacco use is defined in this sensitivity analysis as past-month cigarette smoking. Cannabis use is defined as past-month use.

^2^ According to the World Health Organization Composite International Diagnostic Interview Short-Form (CIDI-SF) (Kessler et al., 1998; Léon et al., 2023).

^3^ Unhealthy alcohol use was defined as an AUDIT-C score ≥3 for women, and ≥4 for men (Bush et al., 1998).

**Supplementary Table 4. Factors associated with tobacco and cannabis co-use (sensitivity analysis, multivariable multinomial regression, with tobacco mono-use and cannabis mono-use as the reference groups, respectively, weighted analyses, n=18,036)**

|  | **Tobacco and cannabis co-use vs. tobacco mono-use**^1^ | | **Tobacco and cannabis co-use vs. cannabis mono-use**^1^ | |
| --- | --- | --- | --- | --- |
| **Sex** | **aOR [95% CI]** | **p-value** | **aOR [95% CI]** | **p-value** |
| Men | 2.7 [2.2-3.3] | <0.001 | 0.9 [0.5-1.6] | 0.796 |
| Women (ref.) | 1 |  | 1 |  |
| **Age** (in years) |  |  |  |  |
| 18-30 | 9.2 [5.8-14.4] | <0.001 | 2.7 [1.0-7.1] | 0.054 |
| 31-44 | 4.6 [2.9-7.4] | <0.001 | 3.3 [1.1-9.6] | 0.028 |
| 45-54 | 2.4 [1.5-4.0] | <0.001 | 3.1 [0.9-11.1] | 0.076 |
| 55-64 (ref.) | 1 |  | 1 |  |
| **Having a job** |  |  |  |  |
| No | 1.5 [1.2-1.9] | <0.001 | 1.6 [0.9-2.7] | 0.117 |
| Yes (ref.) | 1 |  | 1 |  |
| **Educational level** |  |  |  |  |
| <Upper secondary school certificate | 0.9 [0.7-1.1] | 0.330 | 2.5 [1.1-5.5] | 0.021 |
| Upper secondary school certificate | 0.8 [0.6-1.0] | 0.086 | 0.8 [0.5-1.4] | 0.472 |
| > Upper secondary school certificate (ref.) | 1 |  | 1 |  |
| **“Presently, would you say that in your household, financially speaking…?”** |  |  |  |  |
| You are comfortable (ref.) | 1 |  | 1 |  |
| You are ok | 1.1 [0.8-1.4] | 0.610 | 1.6 [0.9-3.0] | 0.131 |
| You just get by | 1.5 [1.1-2.0] | 0.009 | 6.5 [2.6-16] | <0.001 |
| It’s difficult to make ends meet/You can’t manage without going into debt (or using consumer credit) | 1.4 [1.0-2.0] | 0.069 | 3.3 [1.2-9.4] | 0.025 |
| **Major depressive episode^2^** |  |  |  |  |
| No (ref.) | 1 |  | 1 |  |
| Yes | 1.2 [0.9-1.6] | 0.243 | 0.8 [0.4-1.7] | 0.547 |
| **Self-reported health state** |  |  |  |  |
| Very good (ref.) | 1 |  | 1 |  |
| Good | 1.0 [0.8-1.3] | 0.738 | 1.0 [0.6-1.8] | 0.987 |
| Quite good | 1.1 [0.8-1.5] | 0.415 | 2.1 [0.9-4.6] | 0.085 |
| Poor / Very poor | 1.5 [1.0-2.2] | 0.077 | 1.0 [0.3-3.6] | 0.989 |
| **Unhealthy alcohol use^3^** |  |  |  |  |
| No (ref.) | 1 |  | 1 |  |
| Yes | 1.7 [1.4-2.2] | <0.001 | 1.5 [0.8-2.7] | 0.197 |
| **Daily electronic cigarette use** |  |  |  |  |
| No (ref.) | 1 |  | 1 |  |
| Yes | 0.9 [0.7-1.3] | 0.673 | 0.5 [0.3-1.1] | 0.077 |

aOR, adjusted odds ratio; CI, confidence interval.

Data were weighted according to the probability of inclusion, followed by a margin calibration using the sex variable crossed with age in decennial bands, household size, educational qualification, region of residence and size of urban unit (based on 2020 data (Institut National de la Statistique et des Etudes Economiques, 2021a)).

^1^ Tobacco use is defined in this sensitivity analysis as past-month cigarette smoking. Cannabis use is defined as past-month use.

^2^ According to the World Health Organization Composite International Diagnostic Interview Short-Form (CIDI-SF) (Kessler et al., 1998; Léon et al., 2023).

^3^ Unhealthy alcohol use was defined as an AUDIT-C score ≥3 for women, and ≥4 for men (Bush et al., 1998).
